# Implementation and Evaluation of an Economic Model for Telestroke: Experience from *Virtuall*, France

**DOI:** 10.3389/fneur.2017.00613

**Published:** 2017-11-20

**Authors:** Nolwenn Riou-Comte, Gioia Mione, Lisa Humbertjean, Arielle Brunner, Arnaud Vezain, Karine Lavandier, Sophie Marchal, Serge Bracard, Marc Debouverie, Sébastien Richard

**Affiliations:** ^1^Department of Neurology, Stroke Unit, University Hospital of Nancy, Nancy, France; ^2^Agence Régionale de Santé, Grand Est, Nancy, France; ^3^GCS Télésanté Lorraine, Villers-lès-Nancy, France; ^4^Hospital of Bar-le-Duc, Bar-le-Duc, France; ^5^Hospital of Verdun, Verdun, France; ^6^Department of Neuroradiology, University Hospital of Nancy, Nancy, France; ^7^Centre d’Investigation Clinique Plurithématique CIC-P 1433, INSERM U1116, University Hospital of Nancy, Vandoeuvre-lès-Nancy, France

**Keywords:** telestroke, telemedicine, hub-and-spoke model, health economic model, hospitalization costs

## Abstract

**Background:**

Telestroke is recognized as a safe and time-efficient way of treating stroke patients. However, admission centers (spokes) are subject to financial charges which can make them reluctant to join the system. We implemented and assessed an economic model supporting our telestroke system, *Virtuall*, France, which includes one expert center (hub) and six spokes.

**Methods:**

The model is based on payment for the expertise provided by the hub, distribution of charges related to telemedicine according to the fees perceived by the spokes, and transfer of patients between the spokes and the hub. We performed a cost–benefit analysis for all patients included in *Virtuall* from January 2014 to December 2015 to assess the economic balance in each center.

**Results:**

321 patients were prospectively included in the study. Application of the economic model resulted in overall financial balance with funding of a dedicated medical service in the hub, and reduced costs directly related to telestroke by an average of 10% in the spokes. The conditions generating the highest costs for the spokes were: a patient returning from the hub for re-hospitalization (mean cost of $1,995/patient); management of patients treated by intravenous thrombolysis without transfer to the hub (mean cost of $2,075/patient). The most favorable financial condition for the spokes remained simple transfer of patients to the hub and no return (mean cost of $329/patient).

**Conclusion:**

We describe an economic model which can be applied to any telestroke system to ensure the optimal balance between hub and spoke centers.

## Introduction

Telestroke is a safe and time-efficient way of improving access to care for populations geographically remote from competent center (1, 2). Stroke patients are assessed at their nearest hospital (spoke) which is linked to the expert center (hub) to provide clinical examination and cerebral imaging for diagnosis and to deliver intravenous recombinant tissue plasminogen activator (rt-PA) if needed before transfer to a stroke unit (generally located in the hub) (3, 4). This practice improves patient access to reperfusion therapies thereby reducing subsequent disabilities. Telestroke has thus been shown to be cost effective, especially when analysis includes long-term patient outcomes (5). However, the system generates immediate costs which have been poorly assessed to date. Moreover, as these charges are mainly attributed to the spokes, many hospitals are reluctant to become “rt-PA capable” centers representing a hurdle to the implementation of telestroke (6, 7). The answer to this dilemma could be the application of an economic model regulating the financial flow between a hub and its spokes but this has never been reported before. Our telestroke system, *Virtuall* in Lorrain (Grand Est region, France), is supported by a financial agreement between the hub and its six spokes to obtain the best economic balance in all sites. The model is based on payment of tele-expertise, sharing of costs according to the fees perceived by the centers, and the transfer of patients between the hub and the spokes. We conducted a prospective study to assess the resulting economic balance separately for the hub and spokes.

## Materials and Methods

### The Telestroke

Our telestroke system *Virtuall* is based on the hub-and-spokes model. The hub is represented by the stroke unit (department of Neurology) and the department of Neuroradiology of the University Hospital of Nancy, an endovascular thrombectomy capable center. Clinical expertise is ensured by a dedicated 24/7 medical service comprising five neurologists specialized in cerebrovascular diseases. Six spoke hospitals—Bar-le-Duc (A), Verdun (B), Mont-Saint-Martin (C), Sarrebourg (D), Saint-Dié-des-Vosges (E), and Neufchâteau (F)—are connected to the hub *via* a network that allows clinical assessment through audio and video transmission. Cerebral imaging is transmitted for analysis to a neuroradiologist through a teleradiology system. It should be noted that the spoke hospital A became a stroke unit with an intensive care unit on January 1, 2015 but that physicians continue to use the telestroke system for certain diagnostic or therapeutic decisions. Every spoke is able to perform blood tests and cerebral magnetic resonance imaging (MRI). Cerebral computed tomography scans are performed only in cases where MRI is contraindicated.

### The Economic Model

An economic agreement defining the financial flow between the hub and spokes was established for every center enrolled in *Virtuall*. The costs of rt-PA and clinical and radiological tele-expertise are distributed between the spokes and the hub following patient outcome and the fees generated by hospitalization. These fees concern the regular fees perceived by the emergency and medical departments for the spokes, and the regular fees plus supplementary fees (allocated to intensive care units in France) perceived by the stroke unit for the hub.

We identified three scenarios concerning patient management and outcome as follows:

1. Patient admitted to the spoke and transferred to the hub (for specific management and complementary treatment such as endovascular thrombectomy) without returning to the spoke (due to complete recovery, direct transfer to a rehabilitation center, or death).
2. Patient admitted to the spoke and transferred to the hub before returning to the spoke. This return is conditioned by the achievement of several steps in the stroke unit of the hub (control cerebral imaging, etiological exams, first move of the patient from bed to chair, definition of feeding modalities and measures for secondary prevention), and the presence of necessary paramedical staff in the spoke (physio-, ergo-, and speech-therapists).
3. Patients are completely managed in the spoke without transfer to the hub (patients with stroke mimic, transfer refused by the patient or his/her next of kin, agreement between spoke and hub physicians for patients with very poor prognosis, or if there is a lack of available beds in the stroke unit of the hub).

In scenario 1, we consider that the fees perceived by the spoke centers, generated by admission to the emergency department, are so low that rt-PA costs have to be ensured by the hub where fees generated by patient admission in the stroke unit are much higher. Moreover, the charges for neurological and radiological expertise are not allocated to the spokes. In scenarios 2 and 3, we consider that hospitalization of patients in a medical unit generates sufficient fees perceived by the spoke hospitals to cover the cost of rt-PA and expertise from the hub. Whatever the scenario, the hub has to ensure the cost of the dedicated 24/7 medical service. The way the costs and perceived fees are split between the hub and spokes depending on the patient outcome scenario, is described in Table 1.

**Table 1:** Economic model of Virtuall with distribution of costs and perceived fees according to patient outcome scenario.

	Scenario 1				Scenario 2				Scenario 3					
	Spoke		Hub		Spoke		Hub		Spoke		Hub			
	Costs	Fees	Costs	Fees	Costs	Fees	Costs	Fees	Costs	Fees	Costs	Fees		
Cerebral magnetic resonance imaging (USD)	258				258				258				**Overall economic balance**	**Telestroke economic balance**
Urgent blood tests (USD)	53				53				53					
Expertises (USD)	0			0	233			233	233			233		
rt-PA (USD)			1,169		1,169				1,169					
Emergency admission (USD)		26				26				0				
Telemedicine equipment^b^ (USD)	44		31		44		31		44		31			
24/7 medical service^b^ (USD)			86				86				86			
**Balance per patient** (USD)	**−329^a^**		**−1,286^a^**		**−1,731^a^**		**116^a^**		**−1,757^a^**		**116^a^**			
	**−329**		**−117**		**−562**		**116**		**−588**		**116**			
Hospitalization costs^b^ (USD)			7,162		4,604		7,162		4,604					
Hospitalization fees^b^ (USD)				7,114		4,286		7,114		4,286				
**Balance per patient** (USD)	**−329^a^**		**−1,334^a^**		**−2,049^a^**		**68^a^**		**−2,075^a^**		**116^a^**			
	**−329**		**−165**		**−880**		**68**		**−906**		**116**			

*rt-PA: recombinant tissue plasminogen activator*.

*^a^Patient with rt-PA therapy needed*.

*^b^Mean amount per patient; scenario 1: the patient is admitted in the spoke and transferred for hospitalization in the hub without returning to the spoke; scenario 2: the patient is admitted in the spoke and transferred for hospitalization in the hub with return and re-hospitalization in the spoke; scenario 3: the patient is admitted in the spoke without transfer to the hub*.

### Economic Model Evaluation

We performed a multicenter observational prospective study to assess the economic balance of each center involved in *Virtuall* for all patients examined through the system from January 1, 2014 through to December 31, 2015. The following data were collected: clinical data (age, sex, and diagnosis); data about treatment (intravenous rt-PA, endovascular thrombectomy); spoke center of admission; classification in scenario 1, 2, or 3; costs related to admission and hospitalization (for all patient management and healthcare activities in every center including those for medical and paramedical staff, medicines, medical equipment (including for telemedicine and endovascular thrombectomy), consumables, blood tests, imaging, transport, accommodation, laundry, and catering); fees perceived by the spokes due to hospitalization in medical departments for every patient included in scenario 2 or 3, and by the hub due to hospitalization in the stroke unit for every patient included in scenario 1 or 2. Costs concerning telestroke equipment and maintenance were covered by every center through a fixed annual subscription to a telemedicine company calculated on the basis of the center’s activity. Neurologists in the hub received supplementary payment only for acts performed out of usual working hours. Costs due to the dedicated 24/7 medical service were deducted from these remunerations. Hospitalization fees perceived by the centers were assessed from the diagnosis-related group classification [taking into account diagnosis, severity, associated comorbidities, and length of stay (LOS) in hospital] and from a national study about hospitalization costs in France (2013 update) (8).

A first cost–benefit analysis of the telestroke system in every center was performed. It included costs (cerebral imaging, blood tests, neurological and radiological expertise, rt-PA treatment if relevant, telemedicine equipment and the dedicated 24/7 medical service) and perceived fees (admission to the emergency departments for the spokes and expertise for the hub) directly related to patient management through telestroke during the study period.

A second overall cost–benefit analysis in every center was performed. All costs and fees perceived by the centers related to hospitalization of stroke patients in medical departments for the spokes and in the stroke unit for the hub (from admission until discharge or transfer to a rehabilitation center) were added to the first analysis.

Amounts were converted to 2016 US$.

### Ethics

The study was observational without any intervention on human beings. Data were entirely anonymized before treatment. The study received the required legal approval from the appropriate French Protection Committee (Commission Nationale de l’Informatique et des Libertés) (1994826 v 0).

## Results

**Figure 1:**
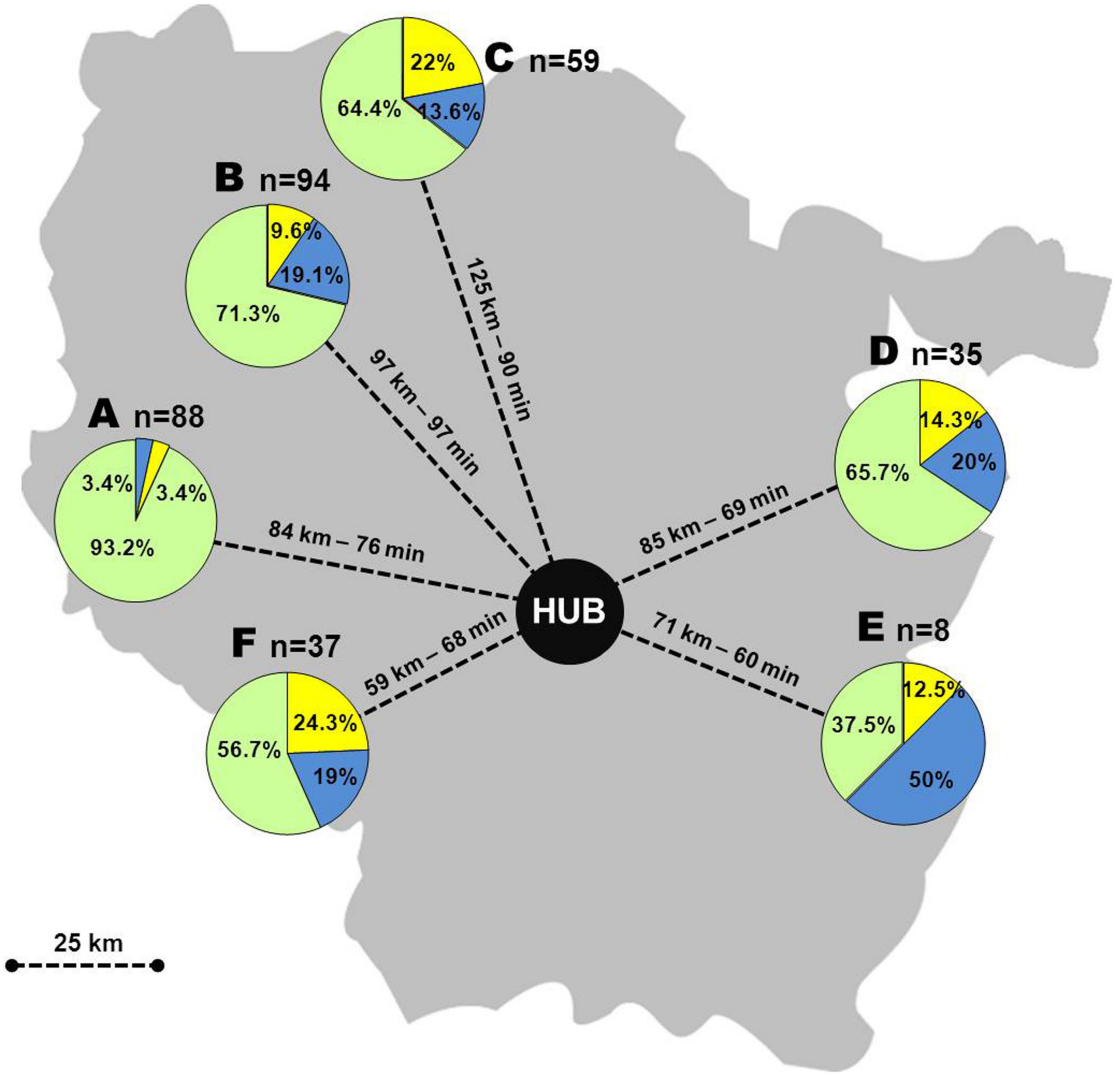
Telestroke Virtuall in the Lorrain region, France, with distribution of patients according to scenarios. A–F: spoke centers, *n*: number of patients, yellow: scenario 1, blue: scenario 2, green: scenario 3.

Overall, 321 patients with an average age of 70 years (range: 23–97 years) and sex ratio (male/female) of 0.84 were included during the study period: 209 patients (65%) presented cerebral infarction; 27 (8%) an intracranial hemorrhage; 21 (7%) transient ischemic attack; and 64 (20%) a stroke mimic. Sixty-seven patients (21%) received intravenous rt-PA and endovascular thrombectomy was performed in three patients after transfer to the hub. The most commonly observed scenario was scenario 3 which accounted for 73% of the patients overall, whereas scenarios 1 and 2 were observed in 15 and 12% of cases, respectively (Figure 1). Overall, 27% of the patients admitted to the spoke centers were transferred to the stroke unit of the hub, which represented 34% of patients diagnosed with an acute cerebrovascular event.

**Figure 2:**
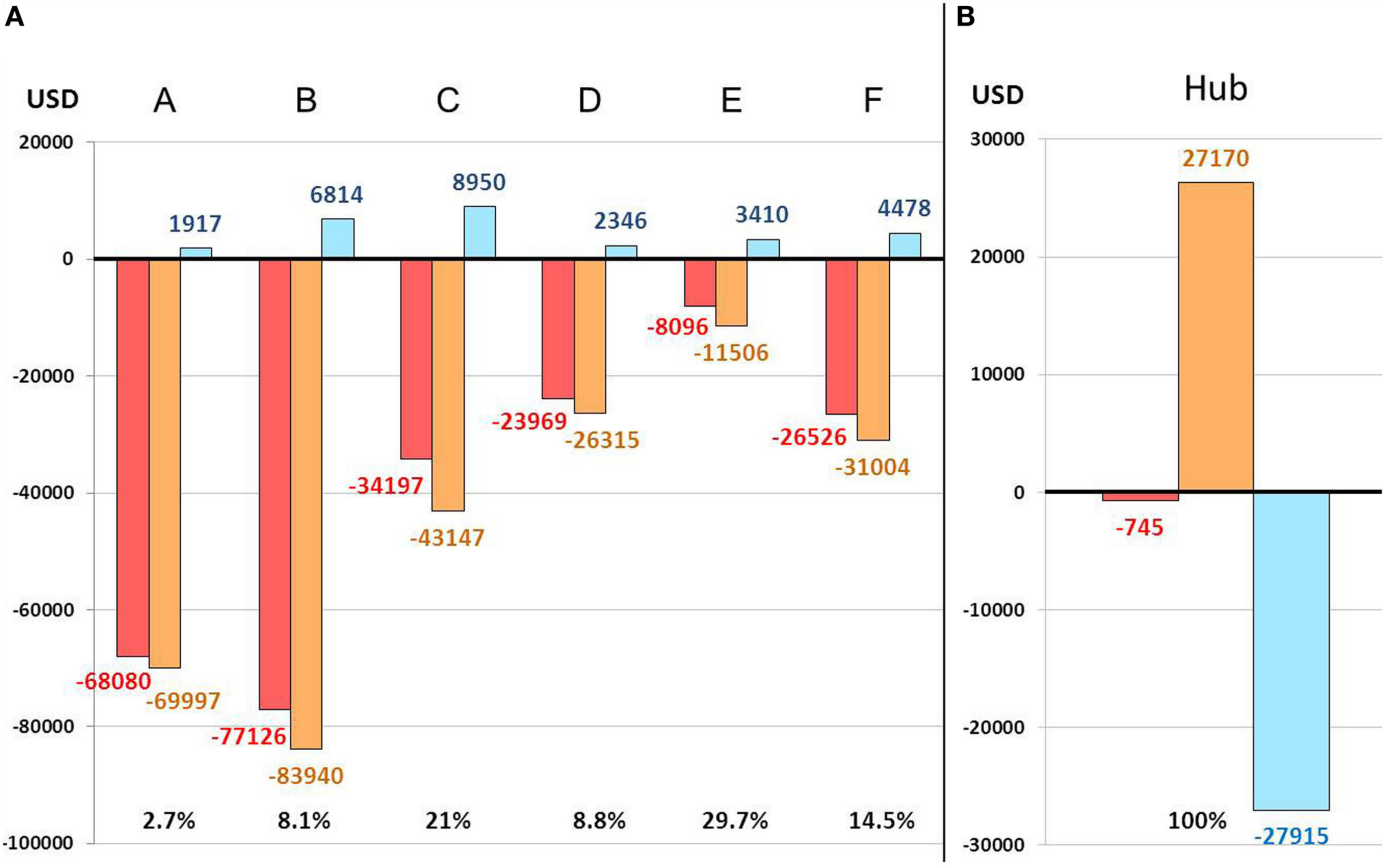
Telestroke economic balance in spokes **(A)** and hub **(B)**. A–F: spoke centers; red: balance observed with application of the economic model; orange: balance estimated without application of the economic model; blue: difference, with percentages, between balance with and without application of the economic model.

Cost–benefit analysis of the telestroke system showed a deficit in all spokes ranging from $8,096 to $77,126. Financial balance was achieved in the hub with a slight deficit of $745. Application of the economic model meant that $27,915 were redistributed from the hub to the spokes. A mean deficit reduction of 10% for the spokes (ranging from 2.7 to 29.7% depending on the center) was estimated by comparing economic balances with the economic model and without as if the model had not been implemented (Figure 2). A per scenario analysis in the spokes showed a mean deficit of $1,731 and $1,757 per patient treated with rt-PA therapy in scenarios 2 and 3, respectively, and $562 and $588 per patient who did not receive rt-PA therapy in the same scenarios. The mean deficit for scenario 1 was $329 per patient whether rt-PA treatment was needed or not (Table 1).

**Figure 3:**
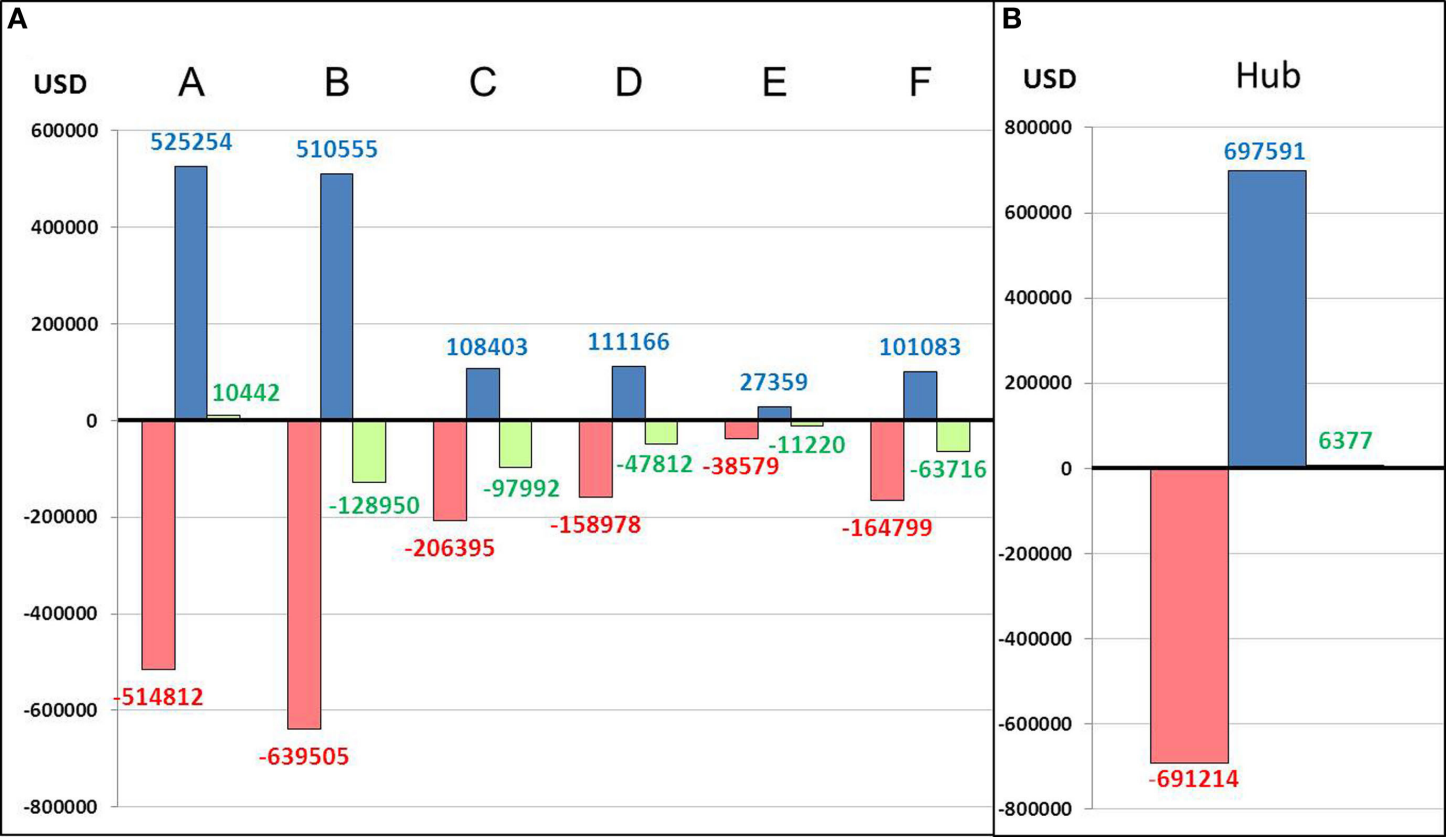
Overall cost–benefit analysis in spokes **(A)** and hub **(B)**. A–F: spoke centers; red: costs; blue: perceived fees; green: balance.

**Figure 4:**
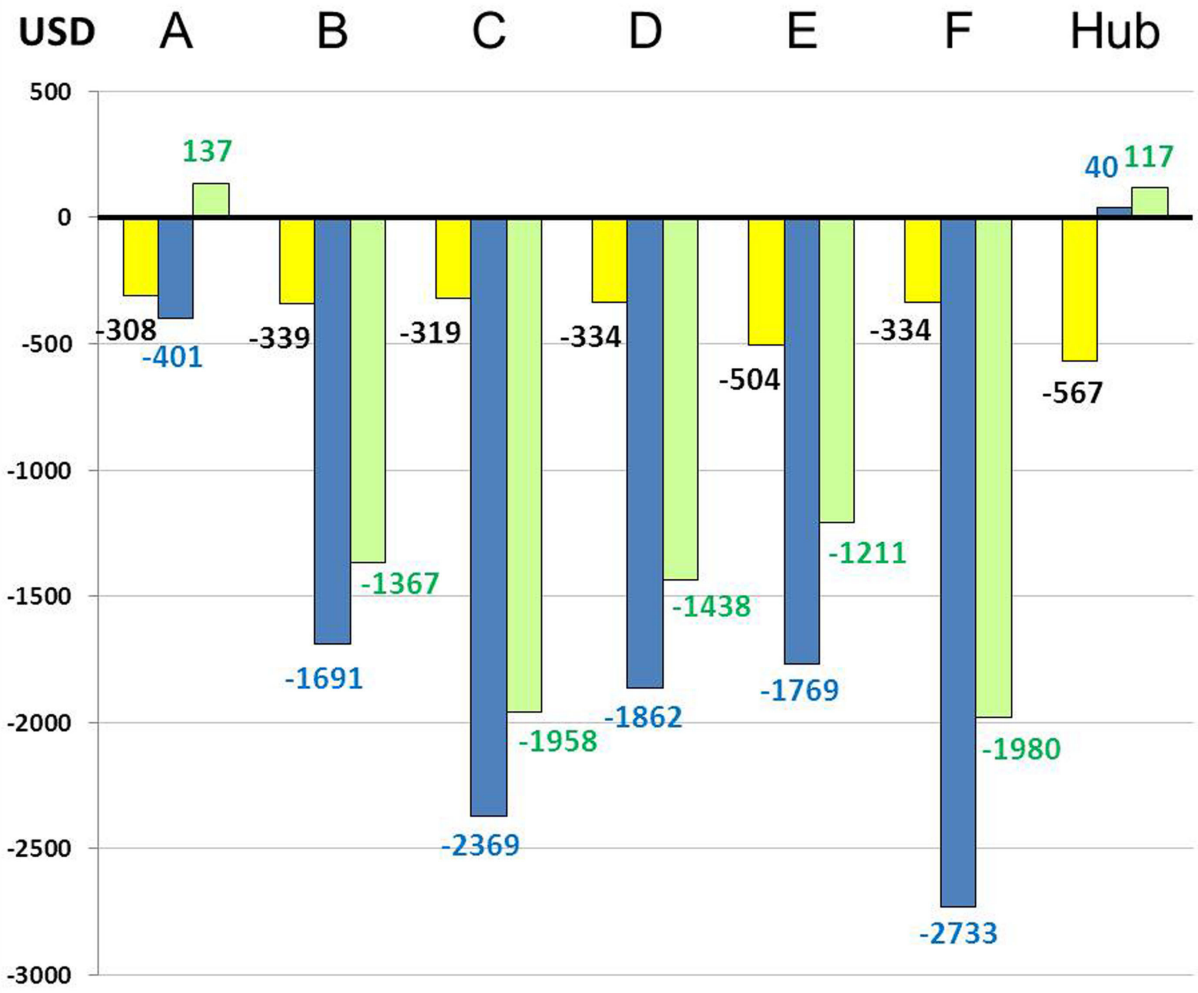
Overall economic balance per scenario and per patient in centers. A–F: spoke centers; yellow: scenario 1; blue: scenario 2; green: scenario 3.

**Figure 5:**
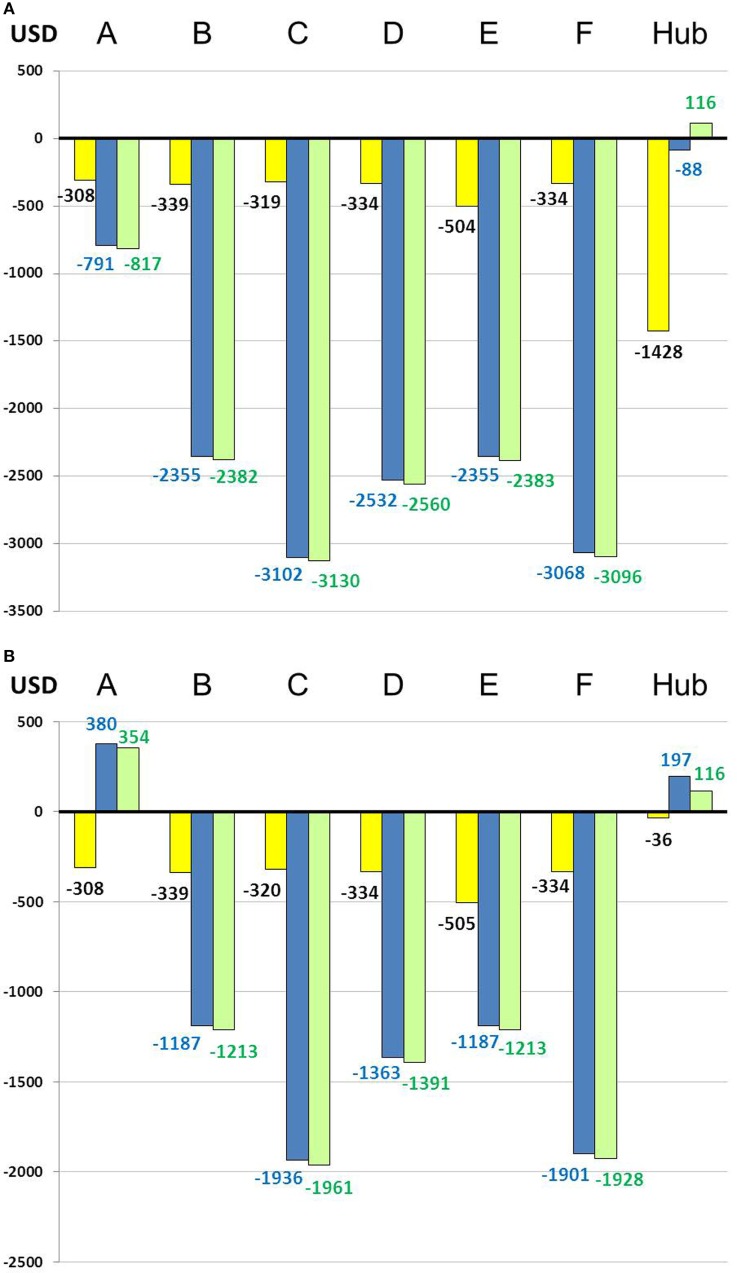
Overall economic balance per scenario and per patient treated with thrombolysis **(A)** and not **(B)**. A–F: spoke centers; yellow: scenario 1; blue: scenario 2; green: scenario 3.

Overall cost–benefit analysis showed slight profit in spoke center A ($10,442) and in the hub ($6,377). Deficits ranged from $11,220 (center E) to $128,950 (center B) in the other spoke centers (Figure 3). Scenario 2 led to the highest mean deficit ($1,995 per patient) in the spokes, whereas the lowest mean deficit ($329 per patient) was observed for scenario 1 (Table 1; Figure 4). The mean deficit for scenario 3 was $992 per patient. The mean LOS in the spokes was 7 days for scenario 2 and 10 days for scenario 3. After dichotomization of patients who received rt-PA or not, the highest mean deficit ($2,075 per patient) for the spokes was found for patients with rt-PA therapy in scenario 3 (Table 1; Figure 5). In the hub, we observed a mean deficit of $567 per patient for scenario 1, and a mean gain of $40 and $117 per patient for scenarios 2 and 3, respectively (Figure 4). The mean LOS in the hub was 9 days for scenario 1 and 5 days for scenario 2. The highest mean deficit ($1,334 per patient) for the hub was found for patients who received rt-PA in the scenario 1 (Table 1; Figure 5).

## Discussion

Our analysis of the economic model implemented for the Virtuall telestroke system (Lorrain, France) demonstrated that the extra costs for the spokes could be efficiently redistributed between spoke and hub depending on patient management and outcome scenarios. This is an important finding as it could encourage more centers to sign up to a telestroke system.

The combination of rt-PA therapy with telestroke to treat stroke patients at the acute phase is a proven strategy to improve long-term prognosis (9). The first major studies about rt-PA therapy estimated a saving of 564 quality-adjusted life years (QALYs) for 1,000 patients treated over 30 years, with a cost-effectiveness calculation of $8,000 saved per QALY (10, 11). Gain in the long-term prognosis was also demonstrated for telestroke with a saving of $2,449 per QALY over a lifetime per patient (12, 13). Nevertheless, this economic gain is at the expense of a financial burden at the acute phase. Recent studies have estimated that the annual cost for a spoke participating in telestroke is €415,000 (14). This includes not only the costs related to the telestroke operation itself but also to the loss of hospitalization fees due to the patient being transferred to the hub. Cost effectiveness for the spokes decreases proportionally to the charges (15). In view of the increasing pressure on hospitals to improve financial management, the costs of any new health-care activity are carefully evaluated before being endorsed. In order to address this issue, we created an economic model to prevent spokes from bearing a high extra financial burden by implementing financial flow between the hub and spokes. Furthermore, implementation of *Virtuall* led to important changes in acute stroke care in our area (an increase in treatment by reperfusion therapies, and transfers from hospitals to stroke unit), the economic consequences of which should be assessed.

The first action was to ensure the hub was paid for the clinical and radiological expertise performed under certain conditions. This resulted in financial balance for the hub and the funding of the specific medical service dedicated to our telestroke system, i.e., five neurologists specialized in cerebrovascular diseases and trained in the management of acute stroke through telemedicine. Overall, the goal was to provide the most rapid and effective support to the spoke hospitals in charge of stroke patients. Moreover, payment for the hub physicians’ clinical and radiological expertise is a way of recognizing their work thereby developing adherence to the system, a crucial element.

The second key point was to split the charges between the hub and spokes according to the fees perceived for each patient. Costs of rt-PA treatment and expertise, which represent a high expenditure, were allocated to the hub rather than the spoke when the patient was permanently transferred to the hub (scenario 1). This led to lower charges for the spokes at a cost of $300 per patient (requiring rt-PA treatment or not), at the expense of a slight deficit for the hub. Distribution of charges between hub and spokes has already been described in literature with a cost effectiveness of $44,804 saved per year and per center (16). We estimated a mean reduction of 10% in the spokes for charges directly linked to telestroke due to our model.

The last concept was to promote the return of the patient from the stroke unit of the hub to the medical department of the initial spoke (scenario 2) so that the spoke perceives the hospitalization fees. This also leads to a shorter LOS in the stroke unit, often conditioned by availability and transfer to a rehabilitation center, and thus to increased patient admissions. Furthermore, after management in the hub, most patients are keen to return to a center near their relatives. Nevertheless, this option proved to be the worst for the spokes in our assessment with the highest deficit per patient. It only resulted in overall economic balance in spoke center A (which became a certified stroke unit during the second year of the study) and the hub which benefited from supplementary fees specific to intensive care units. This latter point is a condition which appears to be essential to achieve financial balance for the hospitalization of a stroke patient. Hospitalization costs are high in the spokes due to the need for specific paramedical staff in centers not originally devoted to stroke patient management. We also hypothesize that patients with the most severe conditions, leading to the highest LOS and costs, were mostly included in this scenario. Moreover, these increased hospitalization fees resulting from patient transfer back to the initial spoke are highly questionable in terms of cost to our healthcare system.

The main issue raised by this last analysis is the cost of hospitalizing a stroke patient. Though estimations from literature vary widely depending on the country where the studies were conducted, hospitalization of a stroke patient represents about half of overall costs related to patient management and is the main cause of economic burden due to stroke (17). Cerebrovascular events are responsible for the highest hospitalization costs among all complications of atrial fibrillation (18). A recent review of published studies found a mean hospitalization cost of $11,635 per patient with cerebral infarction (with $18,543 for studies in United States and $11,900 in Europe) (19). Moreover, an extra charge of $15,000 is observed for patients who receive rt-PA (10, 11). These high costs are also conditioned by patient prognosis and LOS reported as 4.6–12.4 days in literature (17, 20). Higher costs are observed for patients with persistent disability at discharge and inherent increased LOS (20, 21). The only financial answer suggested by literature is to decrease LOS through management in stroke units (11, 22).

The most important global deficit was observed in spoke center B which managed the most patients in the context of scenario 3 (i.e., hospitalization in the spoke without initial transfer to the hub). Furthermore, this is the least favorable for the patient who does not benefit from specialized management in stroke unit leading to increased mortality and LOS (23). This scenario also emphasizes the financial deficit generated in spokes for patients with rt-PA therapy in particular. Finally, it should be reserved for patients for whom the diagnosis of stroke is excluded after tele-expertise leading to rapid discharge from hospital.

Overall, none of the scenarios alone can satisfy both the hub and its spokes. We believe that a combination of the three scenarios, respecting strict rules, results in the best economic balance in every center. Scenario 1 is the best option for all stroke patients with or without rt-PA therapy. This is in conformity with guidelines and the hospitalization fees perceived by the stroke unit compensate the high cost inherent to stroke patient management. Scenario 2 should be applied only for patients with poor recovery potential and high LOS, for whom stroke unit measures are no longer beneficial. Scenario 3, therefore, should be reserved for patients presenting a stroke mimic (migraine, psychiatric disorders, epilepsy …) with no or short hospitalization in the spokes after tele-expertise. Consequently, prioritizing scenario 1 would prevent high hospitalization costs in the spokes, adapted utilization of scenario 2 would help to decrease LOS in the hub, and scenario 3 would generate remuneration fees for the hub to compensate the slight deficit observed in scenario 1.

We recognize limitations to this work. The telestroke *Virtuall* presents particularities that could lead to different results if the economic model were applied in other areas, and even more so, in other countries. Patient transport between the spokes and hub was not charged to the centers but directly paid for by the healthcare system through a specific budget. The emergency department staff could be trained as part of continued professional training which would not lead to a loss of working time. Material for simulation training, as well as the trainers’ time, was provided free of charge by our university center (24). Remuneration of neurologists to ensure the dedicated 24/7 medical service is specific to the French public healthcare system. Costs of rt-PA are underestimated in comparison with other countries. A recent study in United States estimated that it has more than doubled over a period of 10 years ($64.3 per milligram) (25). Moreover, our study included few patients treated with endovascular thrombectomy but we will have to deal with an increasing number of this expensive procedure in future financial evaluations (26). Our study did not include the long-term assessment of patients and the cost savings related to better patient outcomes with fewer disabilities. For instance, analyses did not include costs and fees related to rehabilitation which come under a different charging scheme in France. Nevertheless, we were more interested in the assessment of immediate charges directly attributed to hub-and-spoke centers, and regulated by our economic model. Our results highlight the low percentage of patients transferred to the hub: only one-third of patients with cerebrovascular events were transferred. However, this can partially be explained by the high number of patients admitted to center A which was able to provide most of the expertise of a stroke unit before being officially certified.

To provide further answers, we intend to conduct future cost-effectiveness studies of *Virtuall* to assess the saving of QALYs and related costs. Our economic model is now also applied in the Ile-de-France region which could lead to a new evaluation in a different area that would be of high interest to assess the reproducibility of the model. Since this first assessment, we have observed a markedly improved rate of patients transferred to the stroke unit and an increase in reperfusion therapies (both intravenous thrombolysis and endovascular thrombectomy). Future analyses will include population more representative of international guidelines, thus providing more reproducible results.

## Conclusion

In this economic model for telestroke, distribution of charges between the hub and spokes leads to a reduction in the costs inherent to patient admission and telemedicine in the spokes. Incomes generated by tele-expertise from the hub fund a highly specialized medical service. On the contrary, we failed to demonstrate a benefit to systematically re-admit patients to the spoke hospitals after management in the hub.

## Author Contributions

SR, NR-C, AB, AV, and GM designed the work. NR-C, SR, AV, KL, SM, and GM collected data and drafted results sections, table, and figures. NR-C, SR, LH, AB, GM, MD, and SB conducted the literature review and drafted the background and discussion sections. All authors revised the manuscript critically for important intellectual content and have given their approval of the final submitted version.

## Conflict of Interest Statement

The authors declare that the research was conducted in the absence of any commercial or financial relationships that could be construed as a potential conflict of interest.

## Abbreviations

LOS, length of stay; MRI, magnetic resonance imaging; QALY, quality-adjusted life year; rt-PA, recombinant tissue plasminogen activator.
